# All-trans retinoic acid (ATRA) regulates key genes in the *RARG*-*TOP2B* pathway and reduces anthracycline-induced cardiotoxicity

**DOI:** 10.1371/journal.pone.0276541

**Published:** 2022-11-04

**Authors:** Jafar S. Hasbullah, Erika N. Scott, Amit P. Bhavsar, Erandika P. Gunaretnam, Fudan Miao, Hesham Soliman, Bruce C. Carleton, Colin J. D. Ross

**Affiliations:** 1 Department of Medical Genetics, Faculty of Medicine, The University of British Columbia, Vancouver, British Columbia, Canada; 2 British Columbia Children’s Hospital Research Institute, Vancouver, British Columbia, Canada; 3 Department of Medical Microbiology and Immunology, Faculty of Medicine & Dentistry, University of Alberta, Edmonton, Alberta, Canada; 4 Faculty of Pharmaceutical Sciences, The University of British Columbia, Vancouver, British Columbia, Canada; 5 School of Biomedical Engineering, The University of British Columbia, Vancouver, British Columbia, Canada; 6 Department of Pediatrics, Faculty of Medicine, The University of British Columbia, Vancouver, British Columbia, Canada; Mayo Clinic Arizona, UNITED STATES

## Abstract

The effectiveness of anthracycline chemotherapeutics (e.g., doxorubicin) is limited by anthracycline-induced cardiotoxicity (ACT). A nonsynonymous variant (S427L) in the retinoic acid receptor-γ (*RARG*) gene has been associated with ACT. This variant causes reduced RARG activity, which is hypothesized to lead to increased susceptibility to ACT through reduced activation of the retinoic acid pathway. This study explored the effects of activating the retinoic acid pathway using a RAR-agonist, all-trans retinoic acid (ATRA), in human cardiomyocytes and mice treated with doxorubicin. In human cardiomyocytes, ATRA induced the gene expression of *RAR*s (*RARG*, *RARB*) and repressed the expression of topoisomerase II enzyme genes (*TOP2A*, *TOP2B*), which encode for the molecular targets of anthracyclines and repressed downstream ACT response genes. Importantly, ATRA enhanced cell survival of human cardiomyocytes exposed to doxorubicin. The protective effect of ATRA was also observed in a mouse model (B6C3F1/J) of ACT, in which ATRA treatment improved heart function compared to doxorubicin-only treated mice. Histological analyses of the heart also indicated that ATRA treatment reduced the pathology associated with ACT. These findings provide additional evidence for the retinoic acid pathway’s role in ACT and suggest that the RAR activator ATRA can modulate this pathway to reduce ACT.

## Introduction

Anthracyclines (e.g., doxorubicin) are a class of chemotherapeutics used in cancer treatment [1]. Their ability to inhibit topoisomerase IIα (TOP2A) enzymes present in highly proliferating cells renders them effective against diverse cancer types [1, 2]. However, despite their effectiveness, patients receiving these drugs can experience anthracycline-induced cardiotoxicity (ACT), which can develop as left ventricular dysfunction and lead to heart failure [3–6]. Pharmacogenetic studies have previously identified robust genetic predictors of severe ACT [7–10]. Further research to understand the corresponding biological pathways can lead to additional modes of ACT prevention to improve the safety and efficacy of anthracycline-based cancer therapy.

A genetic variant (rs2229774, Ser427Leu) in *RARG* has been associated with nearly 5-fold increased odds of ACT in three independent pediatric patient populations [7]. RARG belongs to the class of ligand-regulated nuclear receptors termed retinoic acid receptors (RARs) that influence various genomic and non-genomic physiological processes [11]. The genomic effects of RARs primarily involve transcriptional regulation, whereas the non-genomic effects of RARs include activation of kinase cascades such as that of the mitogen-activated protein kinase (MAPK) [11]. RARs utilize their DNA binding domain to recognize DNA sequences known as retinoic acid response elements to regulate expression of specific genes [11, 12]. RARs also have a ligand-binding domain through which RAR ligands such as all-trans retinoic acid (ATRA) can activate their functions [11, 12].

Retinoic acid signaling plays diverse roles in the heart during development, from heart looping, posterior chamber development to ventricular cardiomyocyte differentiation [13–15]. Although the significance of retinoic acid signaling in the heart after development is largely unexplored, recent research points to a role in regulating cardiac remodeling, a process that involves changes in cardiac phenotype after injury [16–18].

RARG is an upstream regulator and repressor of the ACT susceptibility gene topoisomerase IIβ (*TOP2B*) [7, 19]. *TOP2B* is highly expressed in the heart and is required for anthracyclines to promote downstream effects leading to cardiotoxicity [2, 20]. The ACT-associated RARG Ser427Leu variant occurs adjacent to the ligand-binding domain and results in reduced protein function [7, 21]. The consequences of reduced function manifest as reduced ability to activate retinoic acid response elements and reduced repression of *Top2b*, which may lead to increased ACT susceptibility [7]. In line with this hypothesis, recent assessments validate that the cardiomyocytes expressing the RARG Ser427Leu variant are more sensitive to doxorubicin toxicity [21, 22].

Previously, we established that ATRA in the presence of RARG significantly represses *Top2b* expression in rat cardiomyoblasts [7]. We now explore the relationship between ATRA, *RARG*, *TOP2B*, and ACT susceptibility using a human cardiomyocyte and a mouse model of ACT.

## Materials and methods

### Human cardiomyocyte culture conditions

Primary human cardiomyocytes originating from ventricles of the adult heart were purchased from PromoCell (Cat# C-12810), Heidelberg, Germany. Human cardiomyocytes were cultured in myocyte growth medium (PromoCell, Cat# C-22070) supplemented with myocyte supplementation mix and subcultured using DetachKit (PromoCell Cat# C-41210) [23]. The myocyte supplementation mix (PromoCell) is optimized for primary cardiomyocytes and consists of fetal calf serum, recombinant human epidermal growth factor, basic fibroblast growth factor, and insulin. Human cardiomyocytes cultures were maintained in a humidified incubator at 37°C and 5% CO_2._ All experiments were conducted within passages 3 to 10.

### Development of ACT in human cardiomyocytes and ATRA treatments

Serial concentrations of doxorubicin (Doxorubicin hydrochloride, Sigma-Aldrich, St. Louis, MO, USA, Cat# 44583) were used to simulate ACT in human cardiomyocytes. ATRA (Retinoic acid, Tocris Bioscience, Bristol, UK, Cat#0695) was administered as pre-treatment and co-treatment (with doxorubicin) in subdued light conditions.

The ATRA doses 500 nM and 1000 nM used in this study were selected to reflect plasma concentrations which patients in the clinics have safely tolerated. For example, in patients who receive ATRA for acute promyelocytic leukemia (APL) treatment, peak plasma concentrations of ATRA reach approximately 1000 nM [24–26]. Furthermore, ATRA concentrations between 500 nM to 2000 nM induce substantial effects on cellular experiments [24, 27, 28], suggesting that the doses 500 nM and 1000 nM would be suitable for the cellular experiments performed in this study.

### Cell viability assay

Comparative cell survival was measured using a tetrazolium salt (3-(4,5-dimethylthiazol-2-yl)-2,5-diphenyltetrazolium bromide or MTT) based cell viability assay. On day one, 2 x 10^3^ human cardiomyocytes were seeded into each well of a clear 96-well plate in 100 μl of growth medium and cultured overnight. The next day, human cardiomyocytes were pre-treated with ATRA (500 nM, 1000 nM) or the vehicle (DMSO) for 24 hours. The following day, the cells were treated with the respective ATRA dose or the vehicle (DMSO) and serial concentrations of doxorubicin (0 μM, 1 μM, 3.16 μM, 10 μM, 31.6 μM, and 100 μM) for another 24 hours. Cell viability was quantified using MTT assay (Sigma-Aldrich) on POLARstar Omega plate reader (BMG Labtech, Ortenberg, Germany) by measuring absorbance.

### Apoptosis assay

Cellular apoptosis was measured using Caspase-Glo® 3/7 assay (Promega Corporation, Madison, WI, USA, Cat# G8091). Human cardiomyocytes were seeded as described above in an opaque white-walled 96-well plate. The next day, the cells were pre-treated with 500 nM ATRA, 1000 nM ATRA, or the vehicle (DMSO). After 24 hours, the cells were treated with the respective ATRA dose or vehicle (DMSO) and 0 or 10 μM doxorubicin concentration for another 24 hours. The 10 μM concentration of doxorubicin was selected based on the doxorubicin IC_50_ (half-maximal inhibitory concentration) measured in this study and to maintain physiological relevance [29, 30]. The assay was performed according to the manufacturer’s instructions and was read on POLARstar Omega plate reader (BMG Labtech) by measuring luminescence.

### Evaluation of the changes in gene expression in human cardiomyocytes treated with doxorubicin or ATRA

The change in gene expression of selected key ACT response genes was measured upon doxorubicin or ATRA treatment. For this experiment, 2 x 10^5^ human cardiomyocytes were seeded into each well of 6-well plate in 2 mL growth medium [23]. For control and ATRA treatment, the next day and the following day, the cells were treated by replacing the growth medium with vehicle control (DMSO) or ATRA (500 nM). For doxorubicin treatment, the cells were first treated with vehicle (DMSO), and the next day, the cells were treated with doxorubicin (1 μM) and vehicle (DMSO). Cells were collected and stored at -80°C for RNA extraction 24 hours later.

Cells obtained from two independent experiments were pooled together to extract RNA using the PureLink™ RNA Mini Kit (Thermo Fisher Scientific, Waltham, MA, USA). The cDNA was synthesized from 500 ng of the extracted total RNA and used for quantitative PCR (qPCR) in QuantStudio™ 7 Flex Real-Time PCR Instrument (Thermo Fisher Scientific) using TaqMan gene expression assay probes targeting human genes *RARG* (Hs01559234_m1), Retinoic Acid Receptor β (*RARB*) (Hs00977140_m1), Retinoic Acid Receptor α (*RARA*) (Hs00940446_m1), *TOP2A* (Hs01032137_m1), *TOP2B* (Hs00172259_m1), Peroxisome Proliferator-activated Receptor Gamma Coactivator 1-Alpha (*PPARGC1A*) (Hs00173304_m1), Peroxisome Proliferator-activated Receptor Gamma Coactivator 1-Beta (*PPARGC1B*) (Hs00993805_m1), Fas Cell Surface Death Receptor (*FAS*) (Hs00236330_m1), BCL2 associated X, apoptosis regulator (*BAX*) (Hs00180269_m1), Apoptotic Peptidase Activating Factor 1 (*APAF1*) (Hs00559441_m1), Tumor Protein P53 Inducible Nuclear Protein 1 (*TP53INP1*) (Hs01003820_m1), ATP Synthase F1 Subunit Alpha (*ATP5F1A*) (formerly known as *ATP5A1*) (Hs00900735_m1), and Hypoxanthine phosphoribosyltransferase 1 (*HPRT1*) (Hs01003267_m1). Each qPCR reaction consisted of 5 μL 2X TaqMan Universal Master Mix, 0.5 μL TaqMan probe and 2 μL cDNA (1:5 diluted) totaling to 10 μL reaction volume. Standard cycling conditions were maintained. The gene expression assay was performed as three technical replicates for the pooled RNA sample.

### Animal welfare, husbandry, and study design

Six-week-old male B6C3F1/J mice were obtained from The Jackson Laboratory (Bar Harbor, ME, USA, strain #100010) [31]. The mouse model used in this study is based on previous models which recapitulate early characteristic features of ACT [31–34]. After arrival, the mice were housed in a standard controlled environment and had access to food (PicoLab Mouse 20 diet, #5058 LabDiets), water, bedding, shelter, and nesting material. The PicoLab Mouse 20 diet formulated with higher protein content was provided to all mice to counteract the weight loss during doxorubicin and ATRA treatments (S1 Fig).

The mice were given at least a week to acclimatize before the study. All animal work was performed adhering to the Canadian Council on Animal Care guidelines and ARRIVE guidelines [35]. The University of British Columbia Animal Care Committee (protocols: A17-0081, A21-0208) and The University of British Columbia Risk Management Services approved all procedures. A total of fifteen mice were housed as five animals per cage, and each cage was randomized using a computer-based random order generator and assigned to treatment groups (placebo pellet + saline: control, placebo pellet + doxorubicin: doxorubicin-only, ATRA pellet + doxorubicin: ATRA + doxorubicin). No animals were excluded from the study.

This study was performed using a small sample size to gather preliminary evidence on the effect of ATRA on doxorubicin-induced cardiotoxicity. Sample size calculation revealed that five mice per group are sufficient to detect an estimated 5% change in fractional shortening with a power of 80% significance level of 0.05 using the standard deviation of 2.5–2.7% based on a previous investigation [33]. The primary outcome measured in this study was: changes in heart function measured by echocardiography (fractional shortening and ejection fraction). The secondary outcomes of this study were: cardiomyocytes with vacuolated cytoplasm, Terminal Deoxynucleotidyl Transferase dUTP Nick end Labeling (TUNEL) positive cardiomyocytes, fibrosis, edema, hemorrhage, and inflammation in the cardiac tissue sections.

### ATRA/Placebo pellet implantation in mice

The mice were subcutaneously implanted with 5 mg, 60-day release pellets of ATRA (SV-111) or placebo (SC-111) at the lateral side of the neck under isoflurane anesthesia (Innovative Research of America, Sarasota, FL, USA). The implantation site was shaved and cleaned using 2% chlorhexidine soap, followed by 70% isopropanol to implant the pellet. Then, the drug pellet was injected using a precision trocar after the skin was completely dry (MP-182, Gauge 10, Innovative Research of America). The investigator implanted the pellets was blinded to the treatment groups.

### Development of ACT in mice

Doxorubicin treatments were initiated three days after ATRA/placebo pellet implantation (S1 Fig). The mice (7 to 8-weeks old) received intraperitoneal injections of 3 mg/kg doxorubicin (doxorubicin hydrochloride, TEVA Canada limited, Toronto, ON, Canada) or an equivalent saline volume [31–33]. Each treatment group received weekly treatments of doxorubicin (cumulative dose of 24 mg/kg) or saline for eight weeks. Investigators could not be blinded to the treatment groups due to the color of doxorubicin. In addition, there were also apparent differences in the body weight of doxorubicin and saline-treated groups (S1 Fig).

### Assessment of left ventricular cardiac function by echocardiography

Echocardiography was performed on saline-treated (control), doxorubicin-only treated and ATRA + doxorubicin treated mice to evaluate cardiac systolic function (fractional shortening, ejection fraction) between the groups. Left ventricular fractional shortening and ejection fraction were evaluated by transthoracic echocardiography (Visual Sonics Vevo 2100 systems, Fujifilm Visualsonics, Toronto, ON, Canada). Echocardiography measurements were acquired during isoflurane anesthesia at baseline (before doxorubicin treatment), and after three, six, and eight weeks of weekly doxorubicin treatments. Parasternal short-axis view (M-mode) measurements were acquired between heart rates 400 to 450 beats per minute at the papillary muscle level (S1 Table). The analysis was performed blinded to the treatment groups using three M-mode images for each measurement.

### Tissue collection

The mice were euthanized one week after the final dose of doxorubicin (at week 9) (S1 Fig). Euthanasia was performed by thoracotomy and surgical heart removal under deep anesthesia (5.0% isoflurane). Then, the heart was briefly washed with saline and sectioned between the midpoint and the apex. The transverse section containing the ventricles was transferred to 4% paraformaldehyde solution (Thermo Fisher Scientific) for histological analysis [31]. The remaining heart tissue was transferred to RNAlater® (Invitrogen, Waltham, MA, USA, Cat# AM7020) and stored according to manufacturer’s instructions for gene expression analysis.

### Histology

Hematoxylin and Eosin (H&E), TUNEL, and Masson’s trichrome histological staining were performed on ventricular heart tissue to visualize vacuolated cytoplasms, apoptosis, and fibrosis which are early morphological changes of ACT in cardiomyocytes (Wax-it Histology Services Incorporated, Vancouver, Canada) [1, 31, 32]. The tissue sections were graded by an independent pathologist blinded to the treatment groups (Providence Health Care, BC, Canada). The pathologist evaluated the tissue sections for the presence of cardiomyocytes with vacuolated cytoplasm, TUNEL-positive cardiomyocytes, fibrosis, edema, hemorrhage, and inflammation.

### Evaluation of gene expression in mouse heart tissues

Heart tissues stored in RNAlater® at -80°C were transferred to Lysing Matrix D and homogenized in FastPrep-24 homogenizer (MP Biomedicals, Santa Ana, CA, USA). Total RNA from the homogenate was extracted using PureLink™ RNA Mini Kit (Thermo Fisher Scientific). Then, cDNA was synthesized from 500 ng of the extracted total RNA and qPCR was performed in QuantStudio™ 7 Flex Real-Time PCR Instrument (Thermo Fisher Scientific). TaqMan gene expression assay probes targeting mouse genes *Rara* (Mm01296312_m1), *Rarb* (Mm01319677_m1), *Rarg* (Mm00441091_m1), *Top2a* (Mm01296339_m1), *Top2b* (Mm00493776_m1), and *Hprt1* (Mm00446968_m1) were used to perform the qPCR. Each qPCR reaction consisted of 5 μL 2X TaqMan Universal Master Mix, 0.5 μL TaqMan probe and 2 μL cDNA (1:5 diluted) totaling to 10 μL reaction volume, maintaining standard cycling conditions.

### Data analysis

All data analysis were performed using GraphPad Prism v7 or v9 (GraphPad, La Jolla, CA, USA). The cell viability and apoptosis activation were normalized to untreated cells in each condition. The dose-response curves were fitted to a non-linear regression (curve fit), log (inhibitor) vs. normalized response model. The relative gene expressions were calculated using *HPRT1* (for human cells) or *Hprt1* (for mouse tissues) as the reference gene employing the 2^−ΔΔCt^ method [36]. The human cardiomyocyte and mouse gene expression data was transformed to log_2_ fold-change to compare the treatment groups.

## Results

### ATRA increases cell viability of human cardiomyocytes exposed to doxorubicin

**Fig 1:**
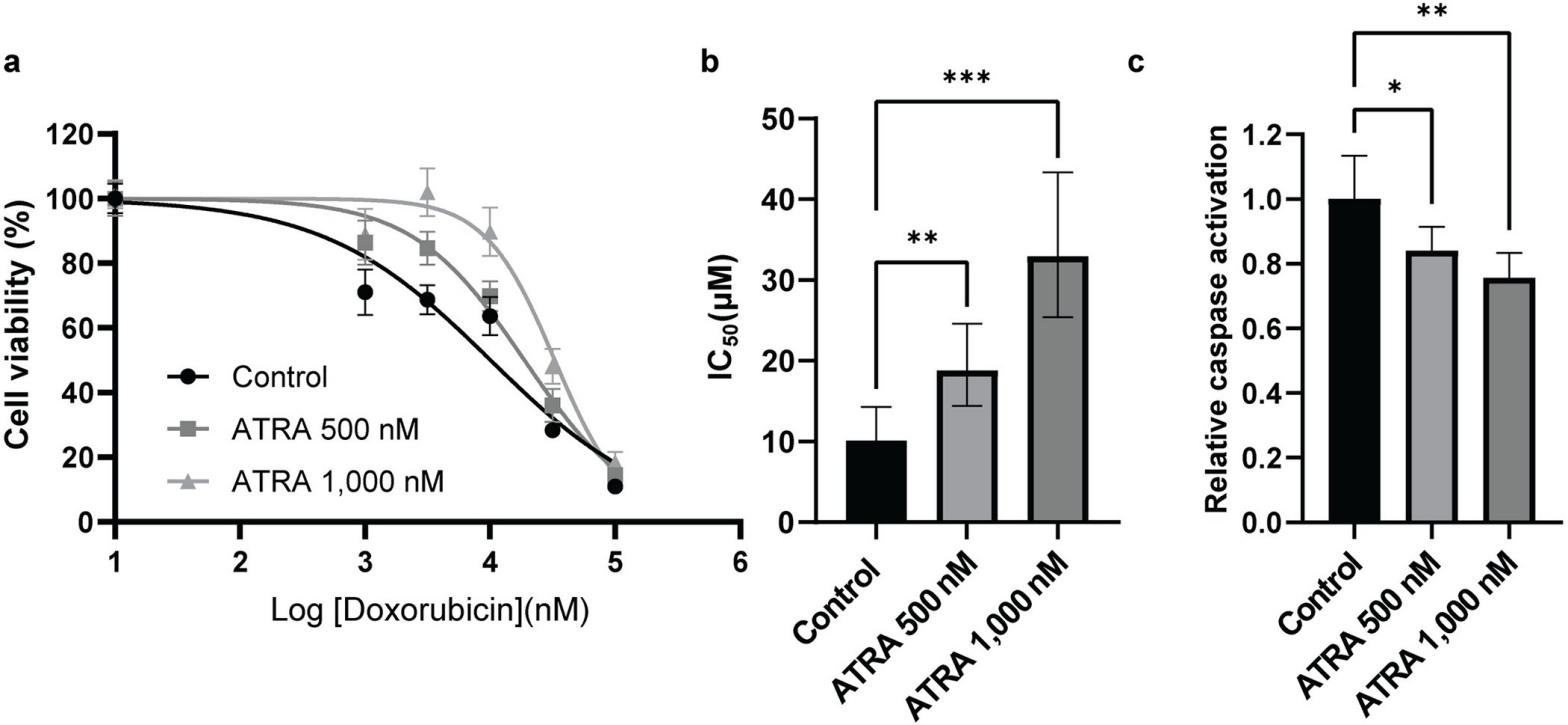
ATRA protects human cardiomyocytes exposed to doxorubicin. (a) Dose-response curves from the MTT cell viability assay. The dose-response curves are plotted for control (vehicle-treated) and ATRA (500 nM, 1000 nM) at serial concentrations (0 μM, 1 μM, 3.16 μM, 10 μM, 31.6 μM, and 100 μM) of doxorubicin (N = 12, 4 independent experiments of 3 biological replicates). Data are presented as mean ± standard error of the mean. (b) IC_50_ values corresponding to each cell-viability curve fit. Data are presented as mean and 95% CI (profile likelihood). Extra-sum-of-squares F test was performed to compare the curve fits (**, and *** denote P = 0.0051 and P < 0.0001, respectively). (c) Relative apoptosis activation measured by caspase 3/7 activity assay. The graph shows apoptosis activation relative to 10 μM doxorubicin vehicle-treated condition. The apoptosis activation was calculated by normalizing to untreated cells in each condition. Relative apoptosis in control (vehicle-treated) and ATRA (500 nM, 1000 nM) at 10 μM doxorubicin treatment is plotted (N = 6 independent biological replicates). Data are presented as mean ± standard deviation. Statistical analysis was performed using one-way ANOVA with Dunnett’s multiple comparisons correction (*, and ** denote P = 0.0250 and P = 0.0013, respectively).

In human cardiomyocytes treated with increasing doxorubicin concentrations, treatment with ATRA increased cell viability compared with vehicle treatment (control) (Fig 1A). Moreover, there was a significant increase in the doxorubicin IC_50_ in the 500 nM ATRA co-treated cells (18.8 μM) and 1,000 nM ATRA co-treated cells (32.9 μM) compared to control (10.1 μM) (control vs. ATRA 500 nM: P = 0.0051, control vs. ATRA 1,000 nM: P < 0.0001, Fig 1B).

Analysis of relative apoptosis activation (caspase 3/7 activation) at 10 μM doxorubicin demonstrated a significant reduction in apoptosis in cells also treated with 500 nM ATRA (16% decrease, P = 0.0250) or 1,000 nM ATRA (24% decrease, P = 0.0013), compared to vehicle-treated cells (control) (Fig 1C).

### ATRA regulates *RAR* genes, *TOP2* genes, and downstream ACT response genes in human cardiomyocytes

**Fig 2:**
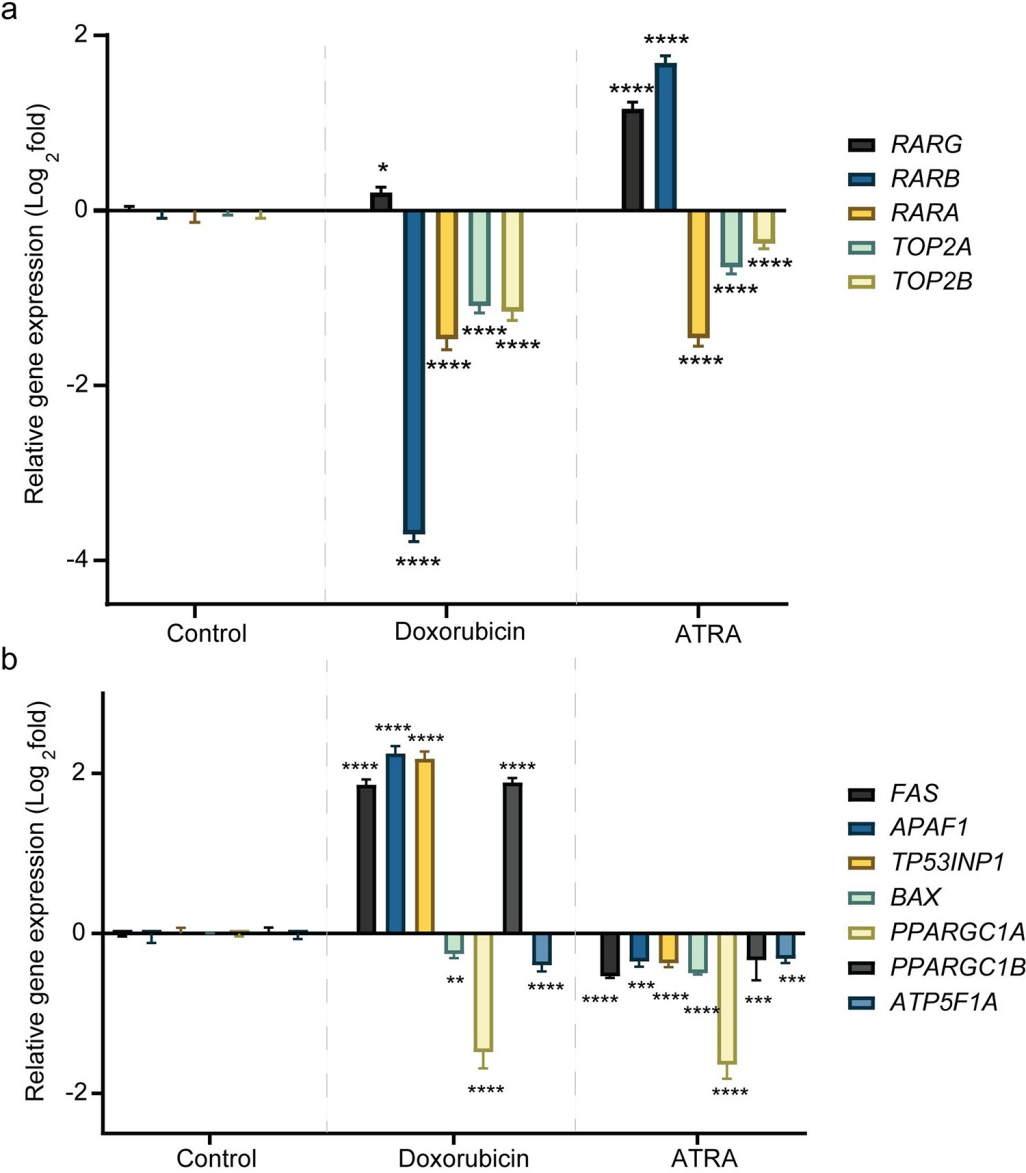
ATRA regulates *RAR* genes, *TOP2* genes, and downstream ACT response genes in human cardiomyocytes. (a) Fold-change in gene expression of *RARG*, *RARB*, *RARA*, *TOP2A* and *TOP2B* during control (vehicle) or doxorubicin (1 μM) or ATRA (500 nM) treatment. Data are presented as mean ± standard deviation (two independent experiments were performed, the extracted RNA was combined, and gene expression assay was performed as three technical replicates, N = 3). Statistical analysis was performed using two-way ANOVA and Bonferroni multiple comparisons correction (comparisons between the control vs. doxorubicin and control vs. ATRA are shown, *, and **** denote P = 0.012, P < 0.0001, respectively). (b) Fold-change in gene expression of the genes involved in doxorubicin-induced apoptosis (*FAS*, *APAF1*, *TP53INP1*, and *BAX*) and doxorubicin-induced mitochondrial dysfunction (*PPARGC1A*, *PPARGC1B*, and *ATP5F1A*) during control (vehicle) or doxorubicin (1 μM) or ATRA (500 nM) treatment. Data are presented as mean ± standard deviation (two independent experiments were performed, the extracted RNA was combined, and gene expression assay was performed as three technical replicates, N = 3). Statistical analysis was performed using two-way ANOVA and Bonferroni multiple comparisons correction (comparisons between the control vs. doxorubicin and control vs. ATRA are shown, **, *** and **** denote, P = 0.0059, P < 0.001 and P < 0.0001, respectively).

In human cardiomyocytes, doxorubicin exposure induces a marginal, but significant increase in *RARG* expression (increased to 0.2-fold, P = 0.012). However, doxorubicin exposure significantly downregulated the expression of *RARB* (decreased to -3.7-fold, P < 0.0001), *RARA* (decreased to -1.5-fold, P < 0.0001), *TOP2A* (decreased to -1.1-fold, P < 0.0001) and *TOP2B* (decreased to -1.2-fold, P < 0.0001) (Fig 2A).

The ACT response genes evaluated are involved in doxorubicin-induced apoptosis and doxorubicin-induced mitochondrial dysfunction [20, 30, 37]. In addition, these genes act downstream of *Top2b*, in cardiomyocytes of a mouse model of ACT [2, 20]. This analysis seeks to understand the effect of these genes upon doxorubicin or ATRA treatment in human cardiomyocytes.

In the downstream ACT response genes evaluated, doxorubicin significantly upregulated the expression of *FAS* (increased to 1.9-fold, P < 0.0001), *APAF1* (increased to 2.2-fold, P < 0.0001), *TP53INP1* (increased to 2.2-fold, P < 0.0001), and *PPARGC1B* (increased to 1.9-fold, P < 0.0001). Further, doxorubicin reduced the expression of the genes *BAX* (decreased to -0.3-fold, P = 0.0059), *PPARGC1A* (decreased to -1.5-fold, P < 0.0001), and *ATP5F1A* (decreased to -0.4-fold, P < 0.0001) (Fig 2B).

The evaluation of relative gene expression in human cardiomyocytes exposed to ATRA versus vehicle control reveals that ATRA significantly induced the gene expression of *RARG* (increased to 1.2-fold, P < 0.0001) and *RARB* (increased to 1.7-fold, P < 0.0001), but repressed the expression of *RARA* (decreased to -1.5-fold, P < 0.0001) compared to vehicle control (Fig 2A). Conversely, ATRA repressed the expression of the genes that encode for the molecular targets of anthracyclines *TOP2A* (decreased to -0.7-fold, P < 0.0001) and *TOP2B* (decreased to -0.4-fold, P < 0.0001) (Fig 2A).

Furthermore, similar evaluation of downstream ACT response genes indicates that ATRA represses the expression of pro-apoptotic genes *FAS* (decreased to -0.5-fold, P < 0.0001), *APAF1* (decreased to -0.3-fold, P = 0.0002), *TP53INP1* (decreased to -0.4-fold, P < 0.0001), and *BAX* (decreased to -0.5-fold, P < 0.0001) (Fig 2B). In addition, ATRA also repressed the expression of genes involved in doxorubicin-induced mitochondrial dysfunction including *PPARGC1A* (decreased to -1.6-fold, P < 0.0001), *PPARGC1B* (decreased to -0.3-fold, P = 0.0004) and *ATP5F1A* (decreased to -0.3-fold, P = 0.0008) (Fig 2B).

### ATRA regulates *Top2a* gene expression in heart tissues of doxorubicin-treated mice

**Fig 3:**
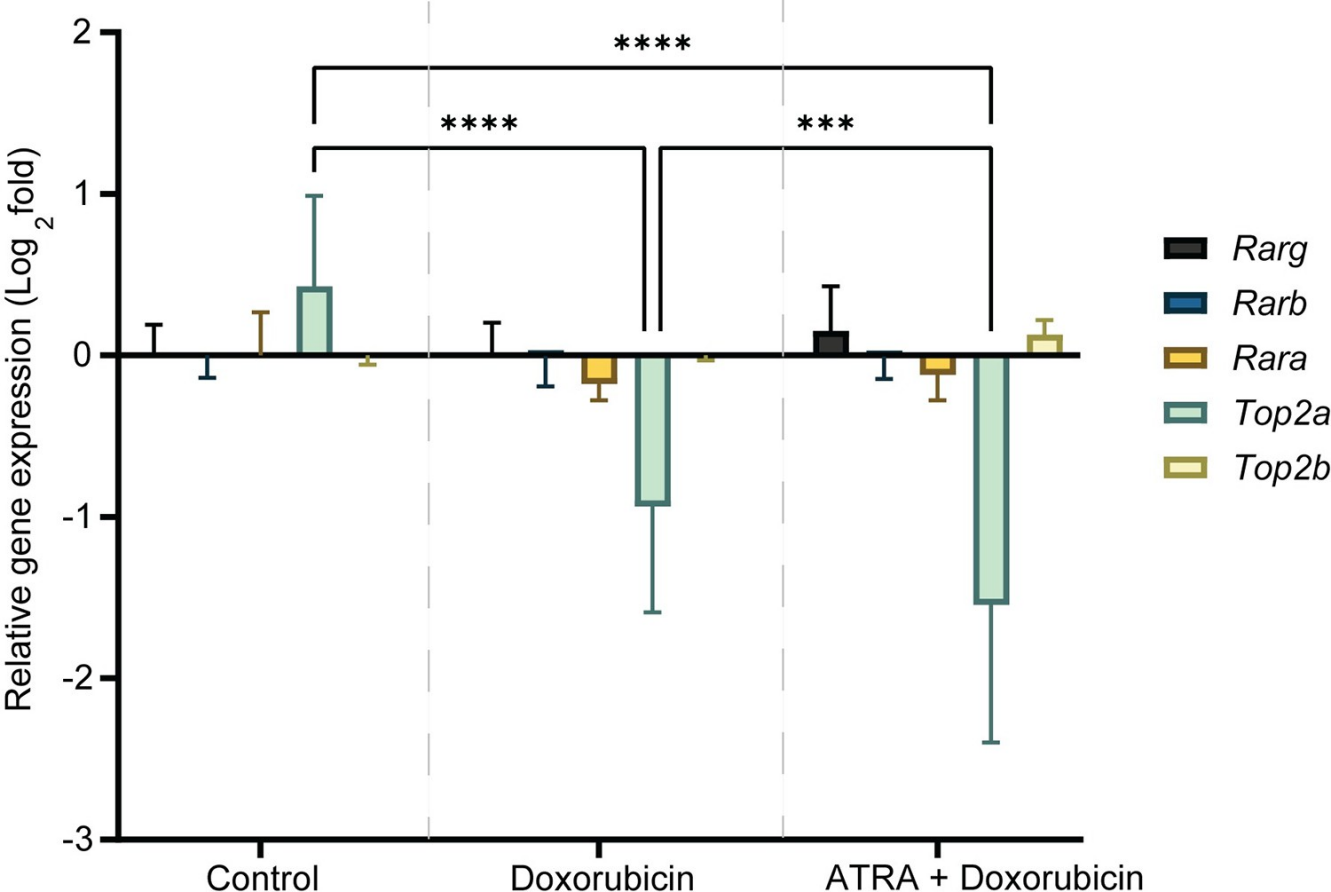
ATRA regulates *Top2a* gene expression in heart tissues of doxorubicin-treated mice. Fold-change in gene expression of *Rarg*, *Rarb*, *Rara*, *Top2a*, and *Top2b* in control (saline-treated), doxorubicin-only treated, and ATRA + doxorubicin-treated mice heart tissues. Data are presented as mean ± standard deviation (N = 9, 3 technical replicates of 3 biological replicates). Statistical analysis was performed using two-way ANOVA and Bonferroni multiple comparisons correction (comparisons between the control vs. doxorubicin, control vs. ATRA + doxorubicin and doxorubicin vs. ATRA + doxorubicin are shown, ***, and **** denote P = 0.0009, P < 0.0001, respectively).

Investigation of the gene expression of *Rar* genes and *Top2* enzyme genes in the mice heart showed that only *Top2a* expression was significantly different in the treatment groups. Compared to control mice, doxorubicin-only treated mice and ATRA + doxorubicin-treated mice showed significant downregulation of *Top2a* expression (doxorubicin-only treated mice: decreased to -0.9-fold, P < 0.0001 and ATRA + doxorubicin-treated mice decreased to -1.5-fold, P < 0.0001). Moreover, the ATRA + doxorubicin-treated group showed significantly reduced *Top2a* expression than the doxorubicin-only treated group (P = 0.0009) (Fig 3).

### ATRA treatment mitigates cardiac systolic dysfunction induced by doxorubicin in mice

**Fig 4:**
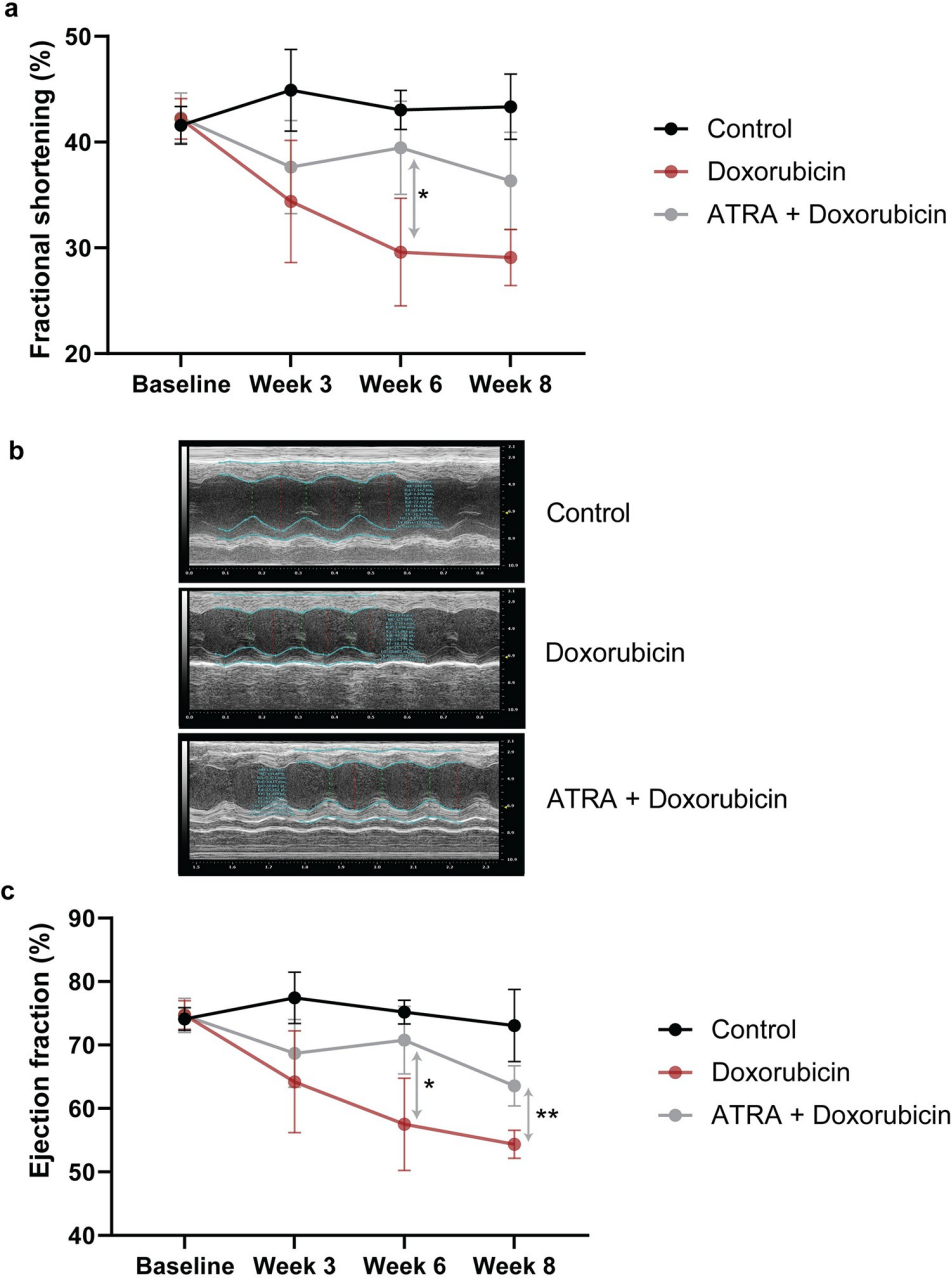
ATRA is cardioprotective in doxorubicin-treated mice as demonstrated by echocardiography. (a) Fractional shortening measurements to evaluate cardiac systolic function in control (saline-treated), doxorubicin-only treated, and ATRA + doxorubicin-treated mice. The graph shows percent fractional shortening, at baseline, after three, six, and eight weeks of weekly doxorubicin/saline treatments (N = 5 mice per group). Data are presented as mean ± standard deviation. Statistical analysis was performed using repeated measures two-way ANOVA with Bonferroni multiple comparisons adjustment (* denotes P = 0.0348). (b) Representative M-Mode echocardiograms in control (saline-treated), doxorubicin-treated, and ATRA + doxorubicin-treated mice after eight weeks of study (cumulative doxorubicin dose of 24 mg/kg in the doxorubicin-treated mice). The y-axis denotes the distance (in mm) from the transducer, and the x-axis denotes time (in ms) [38, 39]. The blue line outlines the left ventricular anterior and posterior walls. The green dotted line represents chamber width during systole, and the red dotted line denotes the chamber width during diastole. (c) Ejection fraction measurements to evaluate cardiac systolic function in control (saline-treated), doxorubicin-only treated, and ATRA + doxorubicin-treated mice. The graph shows percent ejection fraction at baseline, after three, six, and eight weeks of weekly doxorubicin/saline treatments (N = 5 mice per group). Data are presented as mean ± standard deviation. Statistical analysis was performed using repeated measures two-way ANOVA with Bonferroni multiple comparisons adjustment (*, and ** denote P = 0.0193 and P = 0.0020, respectively).

The comparison of percent change in fractional shortening indicates that the percent fractional shortening is similar in all groups at baseline. At week three, the fractional shortening was reduced in both doxorubicin treatment groups (7.8% decline in doxorubicin-only treated mice P = 0.0351 and 4.6% decline in ATRA + doxorubicin-treated mice P = ns/not significant) compared to baseline (Fig 4A), however not statistically significant from each other. By week six, the ATRA + doxorubicin-treated mice exhibited a statistically significant improvement in fractional shortening (9.9%) compared to the doxorubicin-only treated mice (P = 0.0348) (Fig 4A).

The ejection fraction measurements were similar in all the treatment groups at baseline. The ejection fraction measurements also show a similar trend where the percent ejection fraction is reduced in both doxorubicin-treated groups at week three (10.5% decrease in doxorubicin-only treated mice P = ns and 6.0% decline in ATRA + doxorubicin-treated mice P = 0.0025). As observed in the above analysis, at week six, the ATRA + doxorubicin-treated mice presented a significant improvement (13.3%) in ejection fraction compared to the doxorubicin-only treated mice (P = 0.0193) (Fig 4C). Similarly, at week eight, the ATRA + doxorubicin-treated mice exhibited a significant improvement in ejection fraction (9.2%) compared to the doxorubicin-only treated mice (P = 0.002) (Fig 4B and 4C).

### ATRA reduces the histopathological manifestations of ACT in heart tissues of doxorubicin-treated mice

**Fig 5:**
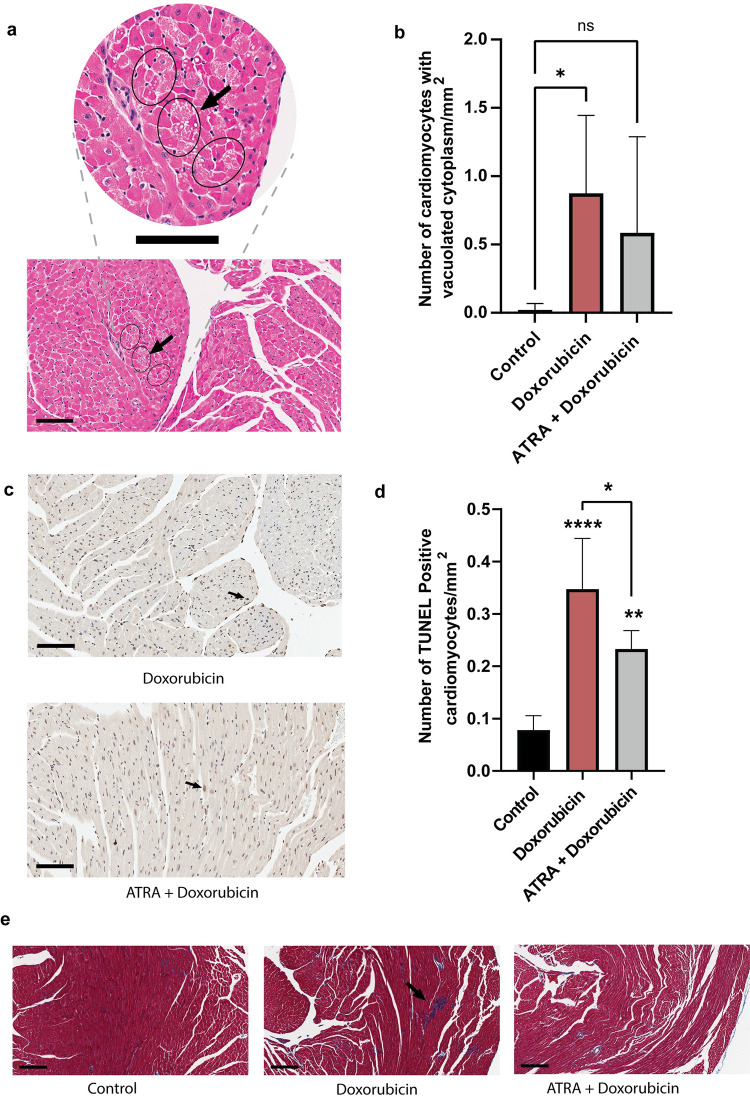
ATRA is cardioprotective in doxorubicin-treated mice as demonstrated by histological analysis. (a) Representative image of cytoplasmic vacuolization in H&E-stained heart tissue section of doxorubicin-only treated mice. The arrow indicates cytoplasmic vacuolization. A zoomed-in view of the cytoplasmic vacuolization is shown on top. Scale bars, 100 μm. (b) Number of cardiomyocytes with vacuolated cytoplasm in the H&E-stained tissue sections of control (saline-treated), doxorubicin-only-treated, and ATRA + doxorubicin-treated mice (N = 5 mice per group). Data are presented as mean ± standard deviation. Statistical analysis was performed using a one-way ANOVA and Dunnett’s multiple comparisons correction (control vs. doxorubicin: *P = 0.0435, control vs. ATRA + doxorubicin: P = ns). (c) Representative image of TUNEL-stained heart tissue sections of doxorubicin-only treated, and ATRA + doxorubicin-treated mice. The arrow indicates TUNEL-positive cardiomyocyte. Scale bars, 100 μm. (d) TUNEL staining to detect DNA fragmentation during apoptosis. The graph depicts the number of TUNEL-positive cardiomyocytes/mm^2^ in the tissue sections of control (saline-treated), doxorubicin-only treated, and ATRA + doxorubicin-treated mice (N = 5 mice per group). Data are presented as mean ± standard deviation. Statistical analysis was performed using one-way ANOVA and Bonferroni multiple comparisons adjustment. Statistical comparisons between the saline-treated condition vs. doxorubicin-treated groups are shown above the bar, comparison between doxorubicin-only treated vs. ATRA + doxorubicin is shown with a bracket (*, **, and **** denote P = 0.0376, P = 0.0055 and P < 0.0001, respectively). (e) Representative images of Masson’s trichrome-stained tissue sections from control (saline-treated), doxorubicin-only treated, and ATRA + doxorubicin-treated mice. The arrow indicates focal fibrosis. Scale bars, 200 μm.

H&E stained sections revealed a 41-fold increase in the number of cardiomyocytes with vacuolated cytoplasms in doxorubicin-only treated mice compared to control mice (saline-treated). Conversely, mice treated with ATRA + doxorubicin exhibited only a 28-fold increase in vacuolated cytoplasms (Fig 5A and 5B). In comparison, the doxorubicin-only treated mice developed significant cardiomyocytes with vacuolated cytoplasms from control (P = 0.0435 vs. control), whereas the mice treated with ATRA + doxorubicin developed 33.1% fewer cardiomyocytes with vacuolated cytoplasm and was not statistically significant from control (P = ns vs. control) (Fig 5B).

TUNEL staining as a measure of DNA fragmentation during apoptosis revealed that both the doxorubicin-only and ATRA + doxorubicin treatments resulted in an increase in the number of TUNEL-positive cells by 4.4-fold and 3-fold, respectively (control vs. doxorubicin: P < 0.0001, control vs. ATRA + doxorubicin: P = 0.0055) (Fig 5C and 5D). However, the ATRA + doxorubicin treatment exhibited a significant protective effect with a 32.9% reduction in the number of TUNEL-positive apoptotic cells compared to the doxorubicin-only treatment (P = 0.0376, Fig 5D).

Histopathological analysis of Masson’s trichrome-stained ventricular heart sections also revealed a protective effect of ATRA in the ATRA + doxorubicin-treated mice. Similar to the control group, the ATRA + doxorubicin-treated mice did not exhibit fibrosis, edema, hemorrhage, or inflammation in any of the sections. In contrast, the sections from the doxorubicin-only treated mice hearts exhibited considerable focal fibrosis (Fig 5E).

## Discussion

The development of debilitating cardiotoxicity and heart failure limits the clinical utility of anthracyclines as mainstay chemotherapeutics. We previously identified a variant in the gene *RARG* (rs2229774, Ser427Leu) that resulted in reduced protein activity [7], which increases susceptibility to severe ACT in patients [7, 21, 22]. In this study, we demonstrate that the administration of a RAR agonist, ATRA, increases *RARG* expression in human cardiomyocytes, presumably with a concomitant increase in RARG activity, and attenuates ACT in both a human cardiomyocyte and a mouse model of ACT.

One of the necessary factors in the mechanism leading to ACT is the cardiac expression of *TOP2B* [20, 40, 41]. We previously demonstrated that ATRA and RARG could repress *Top2b* gene expression in rat cardiomyoblast cells [7]. In this study of human cardiomyocytes, we found that ATRA induces *RARG* and *RARB* expression and represses the expression of the genes that encode for the molecular targets of anthracyclines *TOP2A* and *TOP2B* (Fig 2A). Furthermore, examination of genes that act downstream of the *TOP2B*-mediated ACT susceptibility pathway revealed that genes involved in doxorubicin-induced apoptosis (*FAS*, *APAF1*, *TP53INP1*, and *BAX*) and genes involved in doxorubicin-induced mitochondrial dysfunction (*PPARGC1A*, *PPARGC1B*, and *ATP5F1A*) were also repressed upon ATRA treatment (Fig 2B) [20, 30, 37]. Taken together, these results attest that ATRA transcriptionally regulates RARG mediated ACT response pathway in human cardiomyocytes.

The *RARG* gene where a variant (rs2229774) was associated with ACT susceptibility in patients shares approximately 65% sequence identity with *RARB* and *RARA*. The ability of ATRA to strongly induce *RARB* in human cardiomyocytes suggests that ATRA-mediated cardioprotection may also involve other RARs. Notably, the genes *RARA* and *TOP2A* are situated proximal to each other in opposite strands; similarly, the genes *RARB* and *TOP2B* are located proximal to each other in opposite strands in a different chromosome [21]. The evolutionary conservation of this genomic arrangement across multiple species and the strong regulation of these genes by ATRA suggests that *TOP2* genes are important RAR target genes [21].

In human cardiomyocytes, ATRA significantly induced the gene expression of *RARB*, suggesting a potential role of *RARB* in ATRA-mediated cardioprotection. RARB is involved in the regulation of cell growth, and mutations in *RARB* are associated with cardiac abnormalities [15, 42–44]. Furthermore, selective ligand-mediated activation of RARB2 improved cardiac function in a heart failure model, supporting the notion that RARB could also contribute to ATRA-mediated cardioprotection during ACT [45]. However, future studies are needed to elucidate the specific contribution of *RARB* in the context of ACT.

In parallel to the transcriptional regulation of *RARG* and *TOP2B*, ATRA also enhanced cardiomyocyte survival and reduced apoptosis in human cardiomyocytes exposed to doxorubicin (Fig 1). Furthermore, we also observed the protective effect of ATRA in a mouse model of ACT. Although this was an exploratory study using a small sample size, we detected several early characteristic features of ACT in the doxorubicin-treated groups. In this model, ATRA treatment partially alleviated doxorubicin-induced histopathological manifestations and decline of cardiac function, which are typical repercussions of ACT (Figs 4 and 5) [1, 31–33]. In addition, we also observed strong repression of *Top2a* gene expression in the heart tissues in the doxorubicin treatment group and stronger repression in the ATRA+ doxorubicin treatment group (Fig 3). These findings provide evidence that while reduced functionality of RARG increases ACT susceptibility in patients [7], stimulating RARs using ATRA reduces ACT predisposition in human cardiomyocytes and mice.

In support of our findings, additional reports have shown corroborating evidence that activating the retinoic acid pathway by either retinoic acid precursors or RAR agonists may confer protection against ACT [27, 46–48]. More recently, in a complementary study, Magdy et al. determined that a RARG-specific agonist can similarly mitigate ACT in cells and mice [21]. In that study, by week three the administration of ATRA to mice did not result in a significant reduction in ACT [21]. By contrast, our study examined extended time points and uncovered clear protective effects of ATRA at six and eight weeks of treatment. Notably, our study used a different mouse strain and mode of ATRA administration that may also have contributed to the differences between our study and that reported by Magdy et al.

The mechanisms of ACT susceptibility and ATRA-mediated cardioprotection likely share similar, but opposing, biological pathways. RARs perform various functions and are co-expressed with the ACT susceptibility gene *TOP2B* in the heart. Current evidence suggests that ATRA exerts its protective effect via modulating genomic and non-genomic processes of RARs. As demonstrated in this study, ATRA’s role in regulating the gene expression of *TOP2B* and downstream ACT response genes is an example of the regulation of the genomic process. On the other hand, ATRA’s role in activating the MAPK pathway, specifically the ERK2 (extracellular signal-regulated kinase 2) pathway, can be attributed to the non-genomic process responsible for the protective effect [21, 27]. Further studies to elucidate this mechanism can help identify additional patients at risk of developing ACT due to impairment of this pathway and lead to novel preventative strategies to reduce ACT.

This study demonstrates that ATRA has significant cardioprotective potential to mitigate ACT. RAR agonists are potent activators of various physiological processes and, at high concentrations, can lead to musculoskeletal, neurologic, and teratogenic adverse effects [11, 49]; therefore, it is paramount to consider the safety and efficacy of an retinoic acid based cardioprotectant if advancing to human trials. ATRA has been approved for various indications and has been co-administered together with anthracyclines to patients to treat APL for over two decades [50–53]. The extensive use of ATRA in similar regimens provides years of data on the safety profile of ATRA supplementation in anthracycline-based chemotherapy [54–56].

The clinical use of ATRA in anthracycline-containing chemotherapy regimens provides initial evidence for ATRA’s cardioprotective potential in patients. Studies report a lower incidence of ACT in APL treatments where ATRA is used alongside high cumulative anthracycline doses [51–53, 55–57]. Furthermore, a systematic review and meta-analysis of randomized control trials compared anthracycline-based chemotherapy alone with anthracycline-based chemotherapy plus ATRA in adult acute myeloid leukemia (non-APL) treatment regimens [54]. This meta-analysis showed that the ATRA and anthracycline-containing chemotherapy group developed significantly reduced grade 3–4 cardiotoxicity than the group which received only anthracycline-containing chemotherapy [54]. Notably, ATRA-treated patients developed 56% lower grade 3–4 cardiotoxicity even though patients in both treatment groups received a similar anthracycline dosage [54]. These patient studies suggest that ATRA use with anthracycline-based chemotherapy may result in lower ACT, and future studies could repurpose or optimize ATRA for its cardioprotective properties.

The finding that ATRA represses the *TOP2A* isoform might bring into question the possibility of ATRA interfering with the anticancer efficacy of anthracyclines. On this issue, investigations by Yang et al. and Magdy et al. have shown that ATRA does not interfere with the anticancer effects of doxorubicin using gastric carcinoma and breast cancer cell models, respectively [21, 27]. Furthermore, in the aforementioned systematic review and meta-analysis, the addition of ATRA to anthracycline-containing chemotherapy regimens did not significantly alter overall survival or disease-free survival [54], suggesting that ATRA may not hinder the effectiveness of anthracyclines.

Despite the strengths of our study, we acknowledge that this study also has limitations. First, though the primary human cardiomyocytes were sourced from the ventricular tissue of the adult human heart [23], the cells were proliferative during experimentation. Therefore, there may have been higher *TOP2A* expression than expected in non-proliferative *in vivo* cardiomyocytes. Second, measurement of gene expression levels in the mouse heart tissue after eight weeks of the study shows evidence for ATRA’s role in regulating *Top2a* expression (Fig 3). However, the gene expression of *Rar* genes and *Top2b* were not significantly different between the groups. *RAR* target genes show a dynamic expression pattern after cardiac injury, where gene expressions vary in intensity when measured weeks apart [18]. The reason the *Rar* genes and *Top2b* were not different between the groups in this study could owe to a combination of reasons, including measurement of gene expression at a single time point and measurement of gene expression after weekly doxorubicin and long-term ATRA treatments, at the end of the study [18, 58]. Therefore, future studies could benefit from performing gene expression analyses earlier in the study or at different time points [17, 18, 20]. Third, this study primarily focused on understanding the transcriptional regulation of RAR-response genes in response to ATRA and did not explore the influence of respective proteins. Future studies should address this issue to understand the respective functional and mechanistic contributions of the transcriptional changes.

In summary, this study establishes that the administration of a RAR agonist, ATRA, modulates selected key genes in the *RARG-TOP2B* regulome in human cardiomyocytes and reduces ACT in a human cardiomyocyte and a mouse model of ACT. Further studies are needed to map the branching pathways to understand the breadth of this regulatory network in ACT. In addition, since RARG and TOP2B have direct implications in humans [2, 7], molecular indicators that suggest an impairment in the *RARG-TOP2B* pathway and small molecules that modulate the RARG-TOP2B pathway will also have clinical relevance. Finally, because ATRA modulates the *RARG-TOP2B* pathway, is extensively studied, and has a history of co-administration with anthracyclines in some chemotherapy regimens, ATRA is a suitable candidate cardioprotectant to prevent ACT.

## Supporting information

S1 Fig The study design and change in weight in the mouse model of doxorubicin-induced cardiotoxicity. (a) Experimental outline. ATRA (5 mg/60-day release) or placebo pellet was implanted in mice three days before initiating doxorubicin treatments. From week 1, mice received 3 mg/kg doxorubicin or an equal saline volume every week [31–33]. Echocardiography was performed at week 0, week 3, week 6, and week 8 of study. The mice were sacrificed one week after the last dose of doxorubicin (at week 9), and the heart tissues were collected. (b) Change in body weight over the study period. Percent change in body weight of control (saline-treated), doxorubicin treated, and ATRA + doxorubicin treated mice (N = 5 mice per group). From week 1, the mice received 3 mg/kg doxorubicin or an equal saline volume every week. The starting weight at week 0 was 27.8 ± 1.2 g in the control group, 29.5 ± 1.4 g in the doxorubicin treatment group and 27.3 ± 1.5 g in the doxorubicin + ATRA treatment group. Data are presented as mean ± standard deviation. Statistical analysis was performed using repeated measures two-way ANOVA with Bonferroni multiple comparisons adjustment (*** and **** denotes P = 0.0003 and P < 0.0001, respectively, red* = control vs. doxorubicin, grey* = control vs. ATRA + doxorubicin, doxorubicin vs. ATRA + doxorubicin was not significant in any of the weeks).
(TIF)

S1 Table Summary of heart rates during echocardiography measurements. The echocardiography measurements were acquired between heart rates 400 to 450 beats per minute. No significant differences were found between the groups.
(XLSX)

S1 Data Source data for figures and supplementary figure. (XLSX)
