# Supplementary figures and images for: All-trans retinoic acid (ATRA) regulates key genes in the RARG-TOP2B pathway and reduces anthracycline-induced cardiotoxicity
Source: PLoS One. 2022 Nov 4;17(11):e0276541. doi: 10.1371/journal.pone.0276541 (PMC9635745; doi:10.1371/journal.pone.0276541)

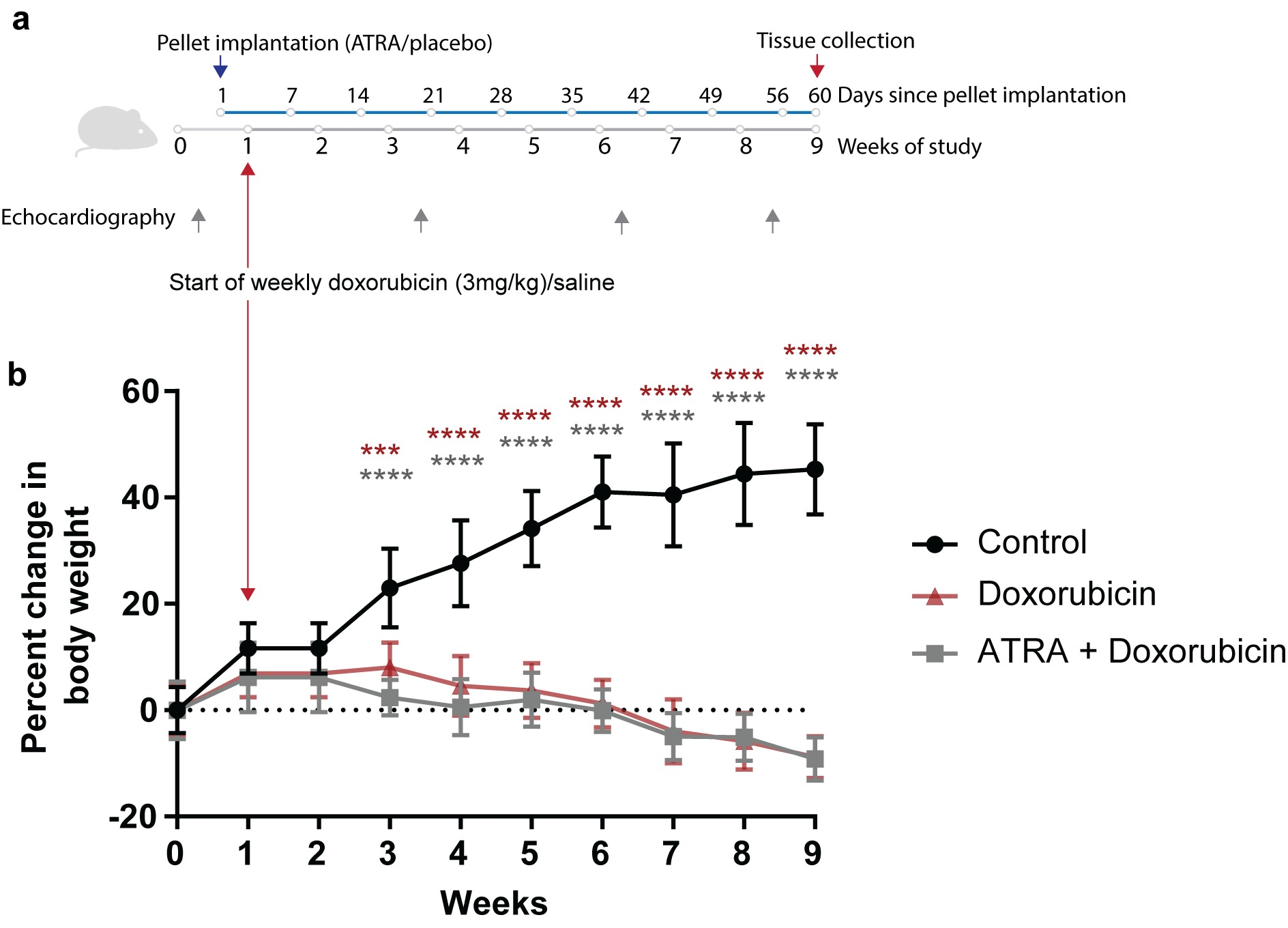

Supplement: S1 Fig — (a) Experimental outline. ATRA (5 mg/60-day release) or placebo pellet was implanted in mice three days before initiating doxorubicin treatments. From week 1, mice received 3 mg/kg doxorubicin or an equal saline volume every week [31–33]. Echocardiography was performed at week 0, week 3, week 6, and week 8 of study. The mice were sacrificed one week after the last dose of doxorubicin (at week 9), and the heart tissues were collected. (b) Change in body weight over the study period. Percent change in body weight of control (saline-treated), doxorubicin treated, and ATRA + doxorubicin treated mice (N = 5 mice per group). From week 1, the mice received 3 mg/kg doxorubicin or an equal saline volume every week. The starting weight at week 0 was 27.8 ± 1.2 g in the control group, 29.5 ± 1.4 g in the doxorubicin treatment group and 27.3 ± 1.5 g in the doxorubicin + ATRA treatment group. Data are presented as mean ± standard deviation. Statistical analysis was performed using repeated measures two-way ANOVA with Bonferroni multiple comparisons adjustment (*** and **** denotes P = 0.0003 and P < 0.0001, respectively, red* = control vs. doxorubicin, grey* = control vs. ATRA + doxorubicin, doxorubicin vs. ATRA + doxorubicin was not significant in any of the weeks). (TIF) [file pone.0276541.s001.tif]
